# The Variations in Care and Real-world Outcomes in Individuals With Rectal Cancer: Protocol for the Ontario Rectal Cancer Cohort

**DOI:** 10.2196/38874

**Published:** 2022-08-05

**Authors:** Sunil Patel, Chad McClintock, Christopher Booth, Shaila Merchant, Carl Heneghan, Clare Bankhead

**Affiliations:** 1 Department of Surgery Queen's University Kingston, ON Canada; 2 Centre for Evidence Medicine University of Oxford Oxford United Kingdom

**Keywords:** rectal cancer, survival, adherence to care, regional variability

## Abstract

**Background:**

Individuals with rectal cancer require a number of pretreatment investigations, often require multidisciplinary treatment, and require ongoing follow-ups after treatment is completed. Due to the complexity of treatments, large variations in practice patterns and outcomes have been identified. At present, few comprehensive, population-level data sets are available for assessing interventions and outcomes in this group.

**Objective:**

Our study aims to create a comprehensive database of individuals with rectal cancer who have been treated in a single-payer, universal health care system. This database will provide an excellent resource that investigators can use to study variations in the delivery of care to and real-world outcomes of this population.

**Methods:**

The Ontario Rectal Cancer Cohort database will include comprehensive details about the management and outcomes of individuals with rectal cancer who have been diagnosed in Ontario, Canada (population: 14.6 million), between 2010 and 2019. Linked administrative data sets will be used to construct this comprehensive database. Individual and care provider characteristics, investigations, treatments, follow-ups, and outcomes will be derived and linked. Surgical pathology details, including the stage of disease, histopathology characteristics, and the quality of surgical excision, will be included. Ethics approval for this study was obtained through the Queen’s University Health Sciences and Affiliated Teaching Hospitals Research Ethics Board.

**Results:**

Approximately 20,000 individuals who meet the inclusion criteria for this study have been identified. Data analysis is ongoing, with an expected completion date of March 2023. This study was funded through the Canadian Institute of Health Research Operating Grant.

**Conclusions:**

The Ontario Rectal Cancer Cohort will include a comprehensive data set of individuals with rectal cancer who received care within a single-payer, universal health care system. This cohort will be used to determine factors associated with regional variability and adherence to recommended care, and it will allow for an assessment of a number of understudied areas within the delivery of rectal cancer treatment.

**International Registered Report Identifier (IRRID):**

RR1-10.2196/38874

## Introduction

Colorectal cancer is the third most common cancer diagnosed in Ontario, Canada, with approximately 9000 cases per year [1]. Ontario (estimated population: 14.6 million) is Canada’s largest province, and residents of Ontario benefit from a single-payer, universal health care system. Diagnostic tests, treatments, and follow-ups for individuals with colorectal cancer are almost exclusively provided through this single-payer system.

The real-world impact of variations in patterns of care and adherence to recommended treatments is an important aspect of determining the effectiveness of a cancer care system. Individuals with rectal cancer in Ontario have an estimated 5-year survival rate of approximately 70% [1], but substantial variations in survival have been noted between regions and hospitals. A previous publication from members of our group found up to an 8% (66.4% vs 58.4%) absolute difference in 5-year overall survival between regions, while also finding up to a 20% (72% vs 52%) absolute difference in 5-year overall survival between the best- and worst-performing hospitals [2]. These differences in outcomes are at least in part due to differences in treatment, access, and the quality of care, as the case mix was minimally different among regions.

The management of individuals with rectal cancer often requires several diagnostic tests, the use of multidisciplinary teams during the delivery of multimodality treatment, and ongoing surveillance for identifying recurrent or metastatic disease. With this level of complexity, the fragmentation of care can occur [3], and inconsistent delivery of treatments may be observed [4]. In addition, due to the nature of the treatments, patients with rectal cancer often experience high rates of short- and long-term morbidity.

There are a number of strategies for assessing the delivery and outcomes of rectal cancer care at a population level. The limitations of many of these approaches are the lack of detailed histopathology data, the inability to assess surgical quality, the discontinuity of care, and high rates of loss to follow-up. The ability to overcome these limitations would substantially improve the ability to assess the real-world effectiveness of cancer care systems and identify the impact of variations in care and/or adherence to recommended care on survival and other important outcomes.

The objective of the Ontario Rectal Cancer Cohort (OntaReCC) is to create a comprehensive clinical-pathological database of individuals who are diagnosed with rectal cancer in Ontario. This database will allow investigators to assess practice patterns, adherence to recommended care, and outcomes within this population. This protocol describes the methods used to establish this database.

## Methods

### Study Setting

Ontario is Canada’s most populous province (estimated population of 14.6 million in 2019) and is a large and diverse region. The province includes large metropolitan areas, as well as sparsely populated rural communities. The demographics of Ontario residents include a wide range of socioeconomic backgrounds, a large number of first- and second-generation Canadians, and indigenous populations [5].

Residents of Ontario are eligible for a government-run, universal, single-payer health insurance plan (ie, the Ontario Health Insurance Plan). This plan covers the costs of physician visits, diagnostic investigations, hospital stays, surgical services, and cancer services (including radiation therapy and chemotherapy). Ontario routinely collects clinical and demographic data, as well as other health care–related data, within a number of linked administrative databases.

### Study Population

Individuals who were diagnosed with rectal cancer between 2010 and 2019 were identified through the Ontario Cancer Registry (OCR) by using the *International Classification of Diseases, Tenth Revision* (ICD-10), codes for rectal cancer (ICD-10 20.9). The OCR captures incident cancer cases through mandatory reporting and is estimated to include >95% of newly diagnosed cancer cases, in which >94% of colorectal cancers were microscopically confirmed [6,7]. Individuals with a histopathology code that was consistent with the adenocarcinoma of the rectum were included. Those aged <18 years at time of diagnosis, those who died on or before the date of diagnosis, and those who were ineligible for the Ontario Health Insurance Plan were excluded. We also excluded those who were diagnosed with cancer at another site (ie, breast cancer, prostate cancer, lung cancer, etc) within the preceding 3 years.

### Data Sources

The OntaReCC combines data from a number of provincially maintained administrative data sets with data from histopathology reports that are made available through the OCR. A full listing of the administrative data sets can be found in Textbox 1. These data sets include health administrative data that are generated whenever a health care service is delivered to an individual, which are utilized for billing, registration, transactions, or record keeping by health care providers. The heath administrative data sets are linked to other provincially maintained data sets that provide vital statistics, census information, and other demographic data.

These data sets provide comprehensive data on individual characteristics (ie, demographics, comorbidities, and socioeconomic status), diagnostic details (the date of diagnosis, histologic variants, and cancer sites), and treatment details. In addition, health care provider details and institution details are captured within these data sets. By using the abovementioned data sets, we will be able to derive important factors or characteristics, including the completeness of preoperative investigations; the type, extent, and completeness of treatments (including surgery, radiation, and chemotherapy); treating clinician characteristics (volumes and practice setting) and institution characteristics (volumes, the level of care, and the presence of a multidisciplinary cancer center); and cancer-related outcomes (overall and cancer-specific survival, the treatment of metastatic disease, the use of palliative treatments, and symptom burden).

**Summary of sources of data to be linked within the Ontario Rectal Cancer Cohort database.**

**Ontario Cancer Registry**

- The Ontario Cancer Registry is the provincial database of information for all Ontario residents who have been diagnosed with cancer (incidence) or have died from cancer (mortality). Data are collected from hospitals, regional cancer centers, pathology reports, and death certificates and cover the entire province of Ontario.

**Cancer Activity Level Reporting database**

- The data elements constitute patient-level activities within the cancer system that focus on radiation and systemic therapy services and outpatient oncology clinic visits. The data set contains clinical, patient-level data.

**Canadian Institute of Health Information-Discharge Abstract Database (CIHI-DAD)**

- The CIHI-DAD captures administrative, clinical, and demographic information on hospital discharges (including deaths, sign-outs, and transfers). This includes demographic, administrative, and clinical data for inpatient discharges (including surgery) and day surgery interventions.

**Ontario Drug Benefit program**

- The Ontario Drug Benefit program provides drug benefits for all adults aged 65 years and older and those receiving social assistance in Ontario. Pharmacists submit claims for each prescribed drug that is covered under the Ontario Drug Benefit formulary. These claims form the basis of the data set.

**Symptom Management Database**

- This database contains data that are used to improve symptom management and collaborative palliative care planning through the earlier identification, documentation, and communication of patient symptoms and performance status.

**Registered Persons Database**

- A listing of all persons who are insured under the Ontario Health Insurance Plan. The data are used to ensure that individuals in other data sources are identified correctly and to support analyses by demographic groups and geography.

### Pathology Data Collection

Histopathology reports were requested from the OCR for individuals who were undergoing biopsy, local excision/polypectomy, or major surgical excision for rectal cancer during the study dates. Cancer Care and Epidemiology at the Queen’s Cancer Research Institute has established processes for abstracting comprehensive details from these types of reports [8-10]. These processes allow for reliable and reproducible data abstraction. A data abstraction manual was created for abstraction. Trained histopathology data abstractors were utilized for data abstraction. Regular meetings for reviewing inconsistencies within and between reports were completed to ensure the validity and reliability of the pathology data abstraction. The data elements are summarized in Textbox 2.

**Histopathology data to be included in the Ontario Rectal Cancer Cohort database.**

**Quality of reporting**

- Synoptic report
- Completeness of reporting

**Quality of excision**

- Intactness of the mesorectum (complete, near complete, or incomplete)
- Margin status (proximal, distal, or circumferential)
- Nodal harvest (≥12)

**Histology**

- Adenocarcinoma and its variants
- Degree of differentiation
- Lymphovascular invasion and perineural invasion

**Stage of disease**

- Tumor stage, nodal stage, and metastatic disease (if applicable)

**Neoadjuvant treatment response**

- Complete response, partial response, or no response (if applicable)

**Mutations and immunohistochemistry**

- *BRAF* and *KRAS* gene status
- Microsatellite instability and mismatch repair proteins

### Exposures and Outcomes

The proposed cohort will contain a number of exposure and outcome variables that are pertinent to a wide range of research questions. Potential exposures include those related to individuals, the circumstances of the cancer diagnosis, health care providers, the institutions where care was delivered, the regions, and the nature and extent of treatments, as summarized in Table 1. A number of outcomes will also be examined and will include the intent and extent of treatments, adherence to recommended care, and short- and long-term outcomes. In addition, symptom burden will be examined, which is a unique feature of the proposed database.

**Table 1:** Summary of the exposures and outcomes that are contained within the Ontario Rectal Cancer Cohort database.

Exposures and outcomes		Variables
**Exposures**		
	Patient characteristics	Age, sex, comorbidities, social economic status, rurality, and geographic region
	Cancer characteristics	Method of diagnosis, symptoms, stage at diagnosis, and histopathologic features
	Treatment	Surgical excision, chemotherapy, and radiation therapy
	Health care providers	Practice setting, specialization, and volume
	Treating institution/hospital	Setting, type, and presence of multidisciplinary care
**Outcomes**		
	Diagnostic and surveillance investigations	Endoscopic, local-regional, and metastatic disease assessments and tumor markers
	Completeness and quality of treatments	Quality of surgical excision, completeness of neoadjuvant and adjuvant therapies, and interruptions in treatments
	Timeliness of care	Diagnosis to investigations, investigations to first treatment, time between treatments, and appropriate timing of surveillance
	Adherence to standard or recommended care	Adherence to staging and treatment and surveillance recommendations based on the stage of disease
	Fragmentation of the delivery of care	Consistent versus inconsistent site of the delivery of surgery, radiation, or chemotherapy
	Utilization of treatments for metastatic disease	Utilization of surgical resection, radiation, and chemotherapy
	Symptom burden	Patient-reported symptoms in various domains and symptom trajectory over time
	Survival	Overall survival and cancer-specific survival

### Ethics Approval

Ethics approval for this research was obtained through the Queen’s University Health Research Ethics Board (approved on October 20, 2020; reference number: 6019418). A number of potential issues were addressed, including how personal health information will be used, the necessity for the linkage of personal health information with other information, and how the linkage would be completed. In addition, we identified potential harms and how they would be mitigated by using previously developed data confidentiality and privacy protocols, via appropriate data handling and curating, via the use of encryption, and via storage on a restricted server behind a firewall. The study will take place at the Queen’s Cancer Research Institute – Cancer Care Epidemiology, which has undergone previous external audits pertaining to data curation and security.

## Results

**Figure 1:**
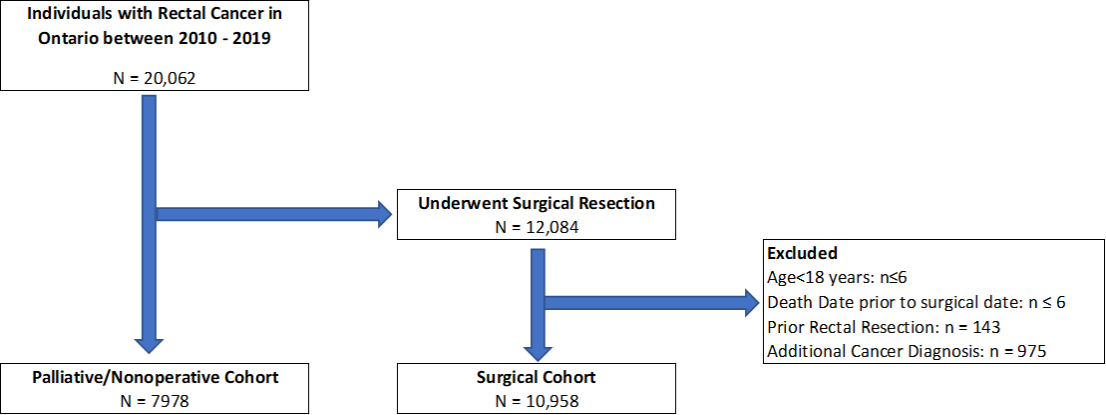
Cohort flow sheet.

We identified approximately 20,062 individuals with a new diagnosis of rectal cancer during the study period. After exclusions, 10,957 unique individuals underwent curative intent surgical resection, while 7980 did not (Figure 1). The average age at the time of diagnosis was 64.9 (SD 12.6) years, with men accounting for 62.8% (6887/10,957) of those included in the surgical cohort. Individuals from a rural setting accounted for 19.3% (2116/10,957), while there was an equal distribution of individuals among income quintiles (lowest quintile: 2193/10,957, 20%; quintile 2: 2248/10,957, 20.5%; quintile 3: 2138/10,957, 19.5%; quintile 4: 2167/10,957, 19.8%; highest quintile: 2148/10,957, 19.6%). Treatment occurred most commonly at a regional cancer center (5914/10,957, 54%), followed by an affiliated cancer center (3626/10,957, 33.1%) and a satellite or nondesignated cancer center (1401/10,957, 12.8%). Adherence to staging investigations, including local-regional and metastatic assessments, occurred in 74.1% (8111/10,957) of individuals. Of the eligible individuals, 62.1% (4148/6681) received radiation therapy, and 58% (3877/6681) received adjuvant chemotherapy.

## Discussion

### Protocol Overview

The overall objective of the OntaReCC is to provide a comprehensive data set of individuals diagnosed with rectal cancer in Ontario, and it will include details on the complete cancer journey (from diagnosis to death). This data set will allow investigators to assess the real-world, population-level outcomes of these individuals and allow for an assessment of the performance of the cancer system. A number of research themes will be explored by using the OntaReCC database, including (1) the regional variability in the delivery of care and outcomes, (2) the predictors and impact of adherence to recommended care, and (3) assessments of other understudied areas of rectal cancer care.

### Regional Variability in the Delivery of Care and Outcomes

Regional variability in survival following the treatment of colorectal cancer has been reported by the Cancer System Quality Index [11]. This report demonstrated a 10% absolute difference in 5-year mortality between the best- and worst-performing regions within Ontario. Other publications identified similar themes [2], demonstrating large variations in 5-year survival between regions and between hospitals despite them having a similar case mix. Members of our group have explored the variation in the delivery of care for individuals with colon cancer. Within this population, significant variations were observed in the use of preoperative investigations [12], surgical quality and pathologic assessments [13,14], and treatment delivery to specific populations [8,9,15,16]. We anticipate similar themes for individuals with rectal cancer, and due to the increased complexity of care, we expect that the magnitude of difference will be greater. The proposed cohort will allow for the assessment of variability in the delivery of care based on geography, hospital type (ie, the presence of a multidisciplinary cancer center), and hospital volume. Importantly, we will use this comprehensive data set to identify predictors of variations in care and outcomes for potential knowledge translation and for interventions at the hospital and regional levels.

### Predictors and Impact of Adherence to Recommended Care

Cancer Care Ontario has developed recommendations for the care of individuals with rectal cancer [17]. These recommendations are consistent with those proposed in other jurisdictions within Canada and elsewhere [18]. Despite the wide availability of clinical practice guidelines and recommendations, significant variability in adherence has been demonstrated in a number of studies [19-24]. The OntaReCC will be utilized to assess the predictors of adherence to recommended care. In addition, we will be able to assess the impact of guideline-adherent care on patient outcomes.

### Assessment of Understudied Areas of Rectal Cancer Care

Practice patterns and outcomes of understudied aspects of rectal cancer care will be assessed, including the treatment of early rectal cancer and end-of-life care.

#### Practice Patterns and Outcomes in Individuals With Early-Stage Rectal Cancer

The OntaReCC will provide an opportunity to explore the practice patterns and outcomes of individuals with early-stage rectal cancer. Although major surgical resection remains the standard of care for many individuals undergoing curative-intent rectal cancer surgery, these procedures have the potential for significant morbidities and poor functional results. In addition, a number of individuals will be required to undergo a permanent colostomy as an outcome of these procedures. Consequently, there has been great interest in avoiding major resection, especially for individuals with early-stage disease. Local excision/polypectomy has been proposed as an adequate alternative for carefully selected individuals [17,25]. Some studies have questioned the safety of this approach [26] and found that careful patient selection is important in ensuring acceptable outcomes. The OntaReCC will allow for the assessment of the appropriateness of local excision (through histopathology data), the risk of local recurrence requiring surgical excision, the risk of metastatic disease, and the impact on overall survival.

#### End-of-Life Care

The type and delivery of care for individuals in their last month of life have been inadequately studied for those with rectal cancer. Our group has assessed potentially aggressive treatments, symptom burden, and the utilization of palliative care services for other disease sites [27-29]. These population-based studies identified the inconsistent delivery of palliative care treatments, high rates of potentially aggressive interventions, and significant symptom burden among many patients. The OntaReCC database will describe variations in end-of-life care and outcomes for this understudied population.

### Strengths and Limitations

As described above, the OntaReCC database will have a near-complete capture of incident rectal cancer cases in Ontario during the study period. Unlike the Surveillance, Epidemiology, and End Results program (around 30% capture) [30] and the National Cancer Database (around 70% capture) [31], the OCR has a near-complete capture of incident colorectal cancer cases [6,7]. As treatment for colorectal cancer is almost exclusively provided through the publicly funded health care system, the linked administrative databases provide a complete description of an individual’s cancer care journey. Low losses to follow-up are expected, regardless of the location or institution in which an individual receives care. By using detailed histopathology data, a comprehensive assessment can be undertaken, which is often not possible with other large population-level data sets or registries. The described cohort will also allow for an assessment of the symptoms that an individual experiences throughout their treatments and follow-ups, which is another unique aspect of the OntaReCC.

Although the proposed cohort will allow for a comprehensive assessment of individuals with rectal cancer, limitations to such population health research exist. First, the reasons behind treatment decisions and patient preferences are unknown. Nonadherence or variations in care may be partially explained by an individual refusing aspects of care or preferring nonstandard approaches, which will not be captured. Second, we will be unable to capture the reasons behind delays in treatment or care, which could be related to nonmodifiable factors. Third, we will not have access to detailed diagnostic radiology reports. This will limit our ability to assess the pretreatment stage of disease (which is primarily determined through magnetic resonance imaging) and the ability to fully capture the development of metastatic disease. Our data set will identify metastatic disease that is either treated (ie, via resection or other local therapies) or documented within applicable linked data sets. Finally, a number of potentially pertinent patient characteristics are not captured well within the available data sets, including individuals’ level of education, employment status, race/ethnicity, obesity, and alcohol or cigarette use.

### Conclusions

The OntaReCC will provide a comprehensive and complete assessment of the care and outcomes of individuals diagnosed with rectal cancer in Ontario. The data set will be an important resource in evaluating the real-world management and outcomes of individuals with rectal cancer. In doing so, we will be able to identify opportunities for improving the delivery of cancer care to this group of individuals.

## Appendix

Multimedia Appendix 1 Peer-review reports from the Canadian Institutes of Health Research / Instituts de recherche en santé du canada (CIHR/IRSC).

## Abbreviations

ICD-10: *International Classification of Diseases, Tenth Revision*
OCR: Ontario Cancer Registry
OntaReCC: Ontario Rectal Cancer Cohort
