# Supplementary material for: The Variations in Care and Real-world Outcomes in Individuals With Rectal Cancer: Protocol for the Ontario Rectal Cancer Cohort
Source: JMIR Res Protoc. 2022 Aug 5;11(8):e38874. doi: 10.2196/38874 (PMC9391972; doi:10.2196/38874)
Supplement: Multimedia Appendix 1 [file resprot_v11i8e38874_app1.pdf]

Review Type/Type d'évaluation: Committee Member 1/Membre de comité 1
Name of Applicant/Nom du chercheur: Patel, Sunil
Application No./Numéro de demande: 428881
Agency/Agence: CIHR/IRSC
Competition/Concours: 2019-10-10 Operating Grant: Data Analysis Using Existing Databases and Cohorts/Subvention de fonctionnement : Analyse de données à partir de bases de données et de cohortes existantes
Committee/Comité: Data Analysis Using Existing Databases and Cohorts/Analyse de données à partir de bases de données et de cohortes existantes
Title/Titre: Disparities in the management and outcomes in patients with rectal cancer: A Population Based Study

---

Assessment/Évaluation:

Purpose: Management of patients with rectal cancer is complex and requires a multidisciplinary approach. Despite existing clinical practice guidelines and a universal, single payer health care system, there is wide variation in survival, and adherence to guidelines for patients with colorectal cancer in Ontario. The Cancer Quality Council of Ontario reports large regional variations in survival, use of adjuvant chemotherapy and use of surveillance colonoscopy. The reasons for these variations have not been explored in a systematic, comprehensive way in patients with rectal cancer.
Hypothesis to be tested or the questions to be answered:
The investigators hypothesize that:
- there is a large variation in receipt of guideline recommended care and that a number of factors are associated with adherence to recommendations
- receipt of guideline recommended care is associated with better survival

Objectives to be achieved and approach proposed:
1) To characterize adherence to guideline recommended care in patients with rectal cancer
2) To identify factors (patient level and provider level) associated with adherence to guideline recommended care
3) To determine whether adherence to guideline recommended care is associated with improved overall survival

Evaluation Criteria

Research Approach: The team will use the existing Ontario Rectal Cancer Cohort Database (ORCC). The database is housed at the division of Cancer Care and Epidemiology (CCE) at the Queens Cancer Research Institute. Database includes 5040 patients who underwent surgical resection for rectal cancer between 2010 and 2015. They will link cohort data to holdings from the Canadian Institute of Health Information (CIHI), the OCR and the Registered Persons Database (RPDB), which are accessible at CCE. They will identify the current state of guideline adherence, factors associated with poor adherence and determine whether receipt of guideline recommended care improves survival in an unselected population.
Originality of the Proposal: Original proposal
Impact of the Research: If hypothesis proves true that guideline adherence improves survival, the results of the study will allow for targeted intervention to improve adherence and ultimately survival for individuals diagnosed with rectal cancer.
Applicant(s): Experienced, well qualified team. Group has completed a number of projects of similar scope and aims.
- PA Assistant Professor, Surgery; limited funding history
- Co-A Booth has funding history as PI with CIHR, Canada Research Chair in Population Cancer Care

Budget: $95,500; Appropriate
Overall Impression: Well written proposal. PA junior but has support from experienced team members.

Review Type/Type d'évaluation: Committee Member 2/Membre de comité 2
Name of Applicant/Nom du chercheur: Patel, Sunil
Application No./Numéro de demande: 428881
Agency/Agence: CIHR/IRSC
Competition/Concours: 2019-10-10 Operating Grant: Data Analysis Using Existing Databases and Cohorts/Subvention de fonctionnement : Analyse de données à partir de bases de données et de cohortes existantes
Committee/Comité: Data Analysis Using Existing Databases and Cohorts/Analyse de données à partir de bases de données et de cohortes existantes
Title/Titre: Disparities in the management and outcomes in patients with rectal cancer: A Population Based Study

---

Assessment/Évaluation:

Synopsis and objectives:

Rectal cancer represents approximately 30% of colorectal cancer cases (with 26,800 new diagnoses in 2017 alone) in Canada; and the management of patients with rectal cancer is complex and requires a multidisciplinary approach. If diagnosed and treated early, rectal cancer have a very good long-term prognosis. Yet, there are regional variations of the benefit of many interventions for rectal cancer, such as adjuvant chemotherapy, neoadjuvant chemoradiation therapy and high quality surgical technique, and hence the variability in survival. The reasons for these variations have not been systematically explored in patients with rectal cancer. Because of the prevalence of rectal cancer and the complexity of management, Cancer Care Ontario (CCO) and other provincial agencies have developed clinical practice guidelines to help clinicians treating rectal cancer patients. These guidelines are to be used to standardize the use of diagnostic tests and treatments with the intent to reduce the risk of recurrence and improve overall survival. So the question being asked is why the persistent variations in rectal cancer outcomes despite the standardized guidelines. This research team therefore hypothesizes that given the existing clinical practice guidelines and a universal, single payer health care system, the variability in the adherence to and receipt of guideline recommended care partially explains the variability in survival within Ontario.

The specific objectives of the proposed research are: to characterize adherence to guideline recommended care in individuals with rectal; identify factors (patient level and provider level) associated with adherence to guideline recommended care; determine whether adherence to guideline recommended care is associated with improved overall survival.

Research approach:
The primary data sources for the proposed study are contained within the Division of Cancer Care and Epidemiology (CCE) at the Queen’s Cancer Research Institute. These data sources include the Ontario Rectal Cancer Cohort, as well as holdings available through the Canadian Institute of Health Information (CIHI), the Ontario Cancer Registry (OCR) and Registered Persons Database. The data sources will be linked via the CCO Group ID to provide a comprehensive data set. These linked data sources will enable examination of management practices and outcomes in rectal cancer patients in Ontario. During the study period, sufficient treatment data is available through linked resources (including diagnostic and treatment data, survival data), and the follow up period is adequate to evaluate our proposed outcomes (survival, surveillance investigations). The proposed analysis plan is appropriate, although I am wondering whether assessing adherence to guidelines can solely be investigated through quantitative data analysis.

Impact
The results of this this research will show the variability in rectal cancer care at the regional level, and

**Review Type/Type d'évaluation:**	Committee Member 2/Membre de comité 2
**Name of Applicant/Nom du chercheur:**	Patel, Sunil
**Application No./Numéro de demande:**	428881
**Agency/Agence:**	CIHR/IRSC
**Competition/Concours:**	2019-10-10 Operating Grant: Data Analysis Using Existing Databases and Cohorts/Subvention de fonctionnement : Analyse de données à partir de bases de données et de cohortes existantes
**Committee/Comité:**	Data Analysis Using Existing Databases and Cohorts/Analyse de données à partir de bases de données et de cohortes existantes
**Title/Titre:**	Disparities in the management and outcomes in patients with rectal cancer: A Population Based Study

Assessment/Évaluation:

therefore point to a process that will allow for directed intervention in improving consistency in care to improve survival at a population level. By addressing variability in adherence to guideline recommended care at the individual and regional levels, outcomes in rectal cancer in Ontario can be improved. The results could be used to develop a comprehensive evaluation of rectal cancer management within Ontario. The findings would result in the identification of current resource utilization, and the adherence with guideline recommended care within out study population.

Applicants

The proposed study is feasible, as this multidisciplinary research team has been using this population level data to evaluate access to care, quality of care, system efficiencies and governance. The team aims to share the findings from this work with all provincial and national organizations to influence health care delivery and policies.

Budget

The budget seems fine, although funds for Queens’ network access seems interesting.

Review Type/Type d'évaluation: SO Notes /Notes de l'agent scientifique
Name of Applicant/Nom du chercheur: Patel, Sunil
Application No./Numéro de demande: 428881
Agency/Agence: CIHR/IRSC
Competition/Concours: 2019-10-10 Operating Grant: Data Analysis Using Existing Databases and Cohorts/Subvention de fonctionnement : Analyse de données à partir de bases de données et de cohortes existantes
Committee/Comité: Data Analysis Using Existing Databases and Cohorts/Analyse de données à partir de bases de données et de cohortes existantes
Title/Titre: Disparities in the management and outcomes in patients with rectal cancer: A Population Based Study

---

Assessment/Évaluation:

Strengths:

• Well-written, comprehensive and original proposal with likelihood of high impact
• Objectives and hypotheses are well-articulated
• Use of numerous linked databases including 5000 pts was a strength
• Well-qualified team with experience in this research realm

Weaknesses:

• The committee was unclear how the applicants will determine whether the guidelines are adhered to for individual patients (this level of methodological detail was generally missing in the protocol)
• The committee asked whether/how the investigators will assess the reasons why guidelines are not adhered to and discussed the notion of qualitative approaches
• How will the applicants take patient choice into account?

Budget:

• No comment
